# Harnessing the extinction vortex against acute lymphoblastic leukemia

**DOI:** 10.1093/emph/eoaf035

**Published:** 2025-11-28

**Authors:** Peng Chen, Sydney Murphy, Huiqing Yeo, Megan Serr, Joel S Brown

**Affiliations:** Genomic Sciences and Systems Biology, Cleveland Clinic Research, Cleveland, OH, USA; Department of Physics, Case Western Reserve University, Cleveland, OH, USA; Department of Microbiology and Molecular Genetics, University of Pittsburgh, Pittsburgh, PA, USA; Faculty of Veterinary Medicine, University of Calgary, Calgary, Canada; Meredith College, Raleigh, NC, USA; Department of Integrated Mathematical Oncology, Moffit Cancer Research Center, Tampa, FL, USA

**Keywords:** acute lymphoblastic leukemia, extinction therapy, extinction vortex, drug resistance

## ACUTE LYMPHOBLASTIC LEUKEMIA

Acute lymphoblastic leukemia (ALL) is the most common pediatric cancer, accounting for $\sim $25% of childhood cancers worldwide [1]. Current treatment employs a 4–6 week **induction** combination therapy, resulting in a complete remission in 95% of patients [2]. This is followed by several months of **consolidation/intensification** using sequences of chemotherapy agents, targeted therapies, and/or immunotherapies tailored to the ALL subtype, and concludes with lower dose **maintenance** therapy for 2–3 years [3]. Since the 1990s [4], the induction and consolidation phases have typically included multiple combinations of therapeutic agents that are switched up after a few weeks or months [3].

## EVOLUTIONARY PERSPECTIVES

The multiphase approach for ALL treatment provides an example of a successful evolution-informed extinction therapy, though not always described in these terms [5, 6]. Outcomes were once poor (long-term remission or cure <10% during 1962–1966) but improved decade by decade to >90% by 2000–2017 (St. Jude therapy studies; Fig. 1 in [3]). This success reflects not only the development of new agents but also the strategy of switching therapies frequently before drug resistance can evolve; simultaneous multi-agent therapy further slows resistance. This is the essence of extinction therapy: apply a set of diverse therapies so a “first strike” (induction) drives a partial or complete response. Then, before resistance can emerge and the cancer rebounds, apply sequences of “second strikes” (consolidation/intensification) that continue to fragment, homogenize, and eliminate small residual cancer cell populations. Each second-strike combination is used long enough to permit efficacy, but not so long as to allow resistance to evolve.

**Figure 1:**
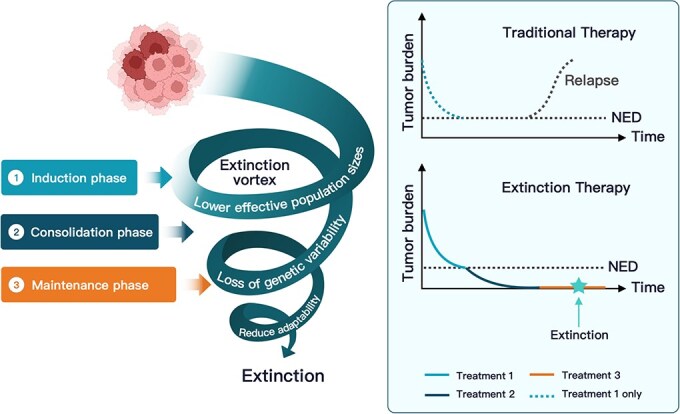
Conceptual depiction of the extinction vortex in ALL treatment, where (Left) induction, consolidation, and maintenance phases impose successive selective pressures that progressively shrink the leukemic population, deplete genetic diversity, and spiral tumor cells into an extinction vortex, and (Right) tumor-burden curves show that single-agent chemotherapy often permits relapse while strategically timed, multi-agent extinction therapy can sustain pressure and achieves tumor eradication.

Extinction therapy draws inspiration from ecological and evolutionary frameworks for preventing species extinctions. The “extinction vortex” [7, 8] describes how the extinction of a species usually entails not just one stressor, such as habitat loss (a first strike), but sequential or tandem additional stressors such as hunting, competition from other species, introduction of invasive species, or loss of genetic diversity (second strikes). This understanding of species extinctions can be flipped to assist in curing what currently are otherwise incurable cancers due to the evolution of drug resistance. An extinction therapy employs sequential and diverse therapies thus subjecting the cancer cells to an array of selection pressures without leaving time for resistance to evolve. These strikes maintain reductions in cancer cell survival and proliferation. As the cancer cell population becomes fragmented, genetic diversity declines due to the loss of alleles and genetic drift. Allee effects may further compromise the fitness of cancer cells in small populations [9]. Together, these processes reduce the ability of the remaining cells to evolve resistance and withstand subsequent stressors.

Treatment of ALL shows a direct connection with the evolutionary and ecological processes underlying the extinction vortex and preventing evolutionary rescue. The first strike drugs (induction phase) often combine cytotoxicity (e.g. Doxorubicin) with mitotic dysregulation of rapidly dividing cells (e.g. Vincristine). Upon remission, second strikes (consolidation/intensification phase) include drugs with different and diverse modes of action, such as Cyclophosphamide (alkylating agent), Methotrexate (inhibitor of purine and pyrimidine synthesis), Etoposide (topoisomerase poison), and others. Continued second strikes (maintenance therapy) maintain selective pressure through lower intensity cytotoxic agents and may add additional modes of action, such as immunotherapy or tyrosine kinase inhibitors, or return to prior drugs such as Vincristine where drug desensitization may have occurred.

Standard of care for many incurable cancers generally follows a different treatment strategy. First-line therapies are not switched until there is measurable disease progression or unacceptable toxicities. With such care, the tumor burden over time tends to follow a U-shaped dynamic where the tumor burden first falls, has a period of remission or stable disease, and then a rise with the evolution of resistance. With extinction therapy, first-line treatment is used as a first strike and, upon a complete response, is immediately switched to second-strike therapies. Under a partial response, another first-strike drug may be used at or before the nadir of tumor burden. This follow-up first strike aims to achieve to no evidence of disease (NED), setting up the subsequent phase of second-strike therapy.

Often guided by mathematical models of cancer dynamics, extinction therapy anticipates resistance and aims to prevent it by proactively switching therapies at critical time points when the tumor population is most vulnerable. This strategy ensures continuous therapeutic pressure, repeatedly diminishing the cancerous population’s adaptability and resilience. Consequently, this approach potentially increases the odds of complete tumor eradication while simultaneously minimizing the cumulative drug exposure and associated toxicities.

## FUTURE IMPLICATIONS

ALL, with its established multiphasic treatment framework and robust monitoring capabilities, represents an ideal candidate for validating the clinical potential of extinction therapy. Extinction therapies hold promise for solid tumors, using blood biomarkers, circulating tumor DNA, and imaging data to guide personalized treatment strategies in real-time. There are presently three non-randomized clinical trials of extinction therapy on pediatric rhabdomyosarcoma (NCT04388839), hormone positive metastatic breast cancer (NCT06409390), and high-risk metastatic castration-sensitive prostate cancer (NCT05189457) [10]. Similar first- and second-strike approaches have been proposed for metastatic Ewing and bone sarcomas (NCT07194044) [11, 12].
